# Publisher Correction: Human neural dynamics of real-world and imagined navigation

**DOI:** 10.1038/s41562-025-02210-9

**Published:** 2025-04-17

**Authors:** Martin Seeber, Matthias Stangl, Mauricio Vallejo Martelo, Uros Topalovic, Sonja Hiller, Casey H. Halpern, Jean-Philippe Langevin, Vikram R. Rao, Itzhak Fried, Dawn Eliashiv, Nanthia Suthana

**Affiliations:** 1 Department of Psychiatry and Biobehavioral Sciences, Jane and Terry Semel Institute for Neuroscience and Human Behavior, University of California Los Angeles, Los Angeles, CA USA; 2 Department of Biomedical Engineering and Department of Psychological and Brain Sciences, Center for Systems Neuroscience, Cognitive Neuroimaging Center, Neurophotonics Center, Boston University, Boston, MA USA; 3 Department of Neurosurgery, Stanford University School of Medicine, Stanford, CA USA; 4 Department of Neurosurgery, Perelman School of Medicine, Richards Medical Research Laboratories, Pennsylvania Hospital, University of Pennsylvania, Philadelphia, PA USA; 5 Department of Neurosurgery, David Geffen School of Medicine, University of California Los Angeles, Los Angeles, CA USA; 6 Neurosurgery Service, Department of Veterans Affairs Greater Los Angeles Healthcare System, Los Angeles, CA USA; 7 Department of Neurology and Weill Institute for Neurosciences, University of California San Francisco, San Francisco, CA USA; 8 Department of Neurology, University of California Los Angeles, Los Angeles, CA USA; 9 Department of Psychology, University of California Los Angeles, Los Angeles, CA USA; 10 Department of Bioengineering, University of California Los Angeles, Los Angeles, CA USA

**Keywords:** Spatial memory, Hippocampus

Correction to: *Nature Human Behaviour* 10.1038/s41562-025-02119-3, published online 10 March 2025.

In the version of the article initially published, Fig. 3c was identical to Fig. 3a and has now been amended with the correct data in the HTML and PDF versions of the article.

