# Supplementary material for: Human neural dynamics of real-world and imagined navigation
Source: Nat Hum Behav. 2025 Mar 10;9(4):781–93. doi: 10.1038/s41562-025-02119-3 (PMC12018265; doi:10.1038/s41562-025-02119-3)
Supplement: Supplementary file 1 — Supplementary Figs. 1 and 2. [file 41562_2025_2119_MOESM1_ESM.pdf]

nature human behaviour

Supplementary information

https://doi.org/10.1038/s41562-025-02119-3

Human neural dynamics of real-world and imagined navigation

In the format provided by the authors and unedited

Supplementary Information

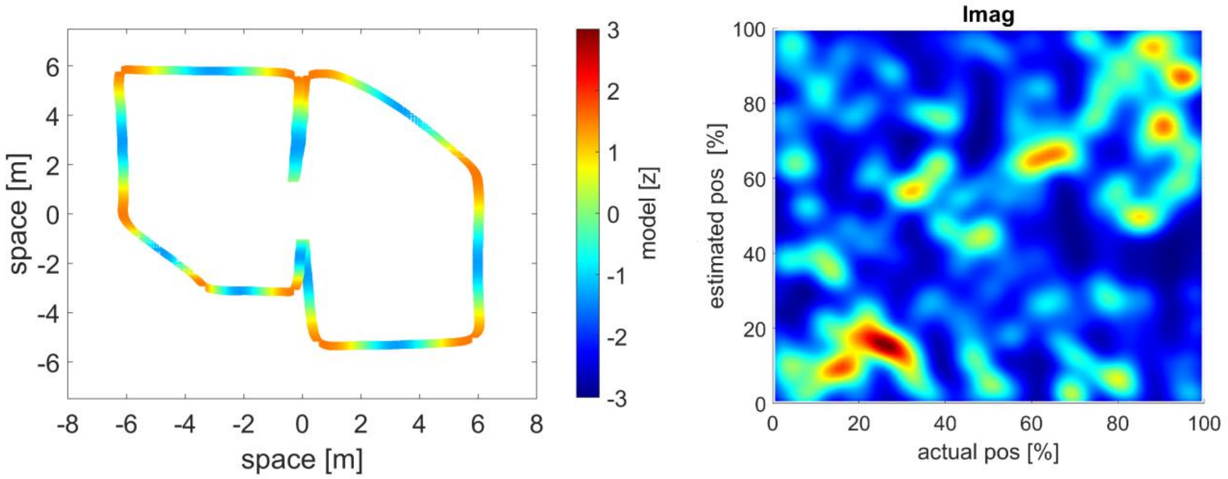

Supplementary Fig. 1 | Modified position reconstruction. The modified cosine route representation for Participant 2 included an additional turn (left panel). Position reconstruction results for the group using this modified model for Participant 2 are shown in the right panel. For comparison, the original model with one fewer turn is shown in Fig. 4g.

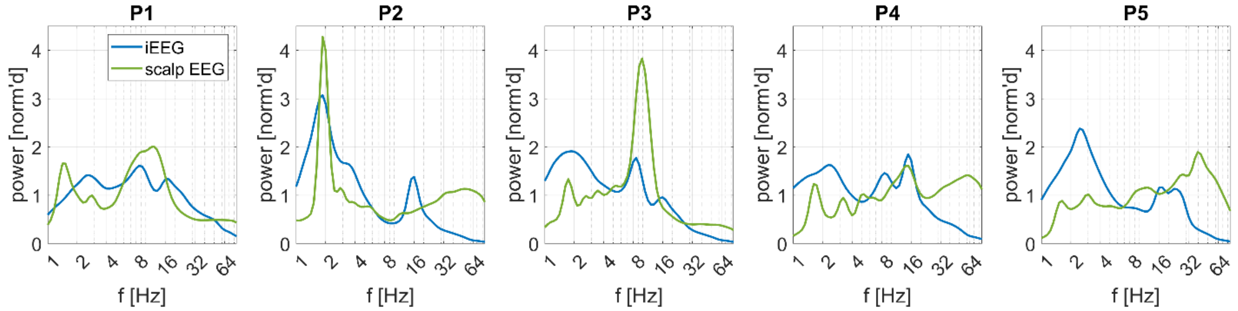

Supplementary Fig. 2 | Preliminary analysis of intracranial and scalp EEG. Frequency spectra from individual participants (P1-P5) show peaks in both intracranial and scalp EEG recordings, with some overlap in the lower frequency ranges. Intracranial EEG recorded from the MTL is plotted alongside averaged scalp EEG recorded from posterior occipital electrodes (PO3, PO4). Future studies will be required to investigate the precise relationship between MTL and scalp EEG activity.
