# Supplementary material for: Human neural dynamics of real-world and imagined navigation
Source: Nat Hum Behav. 2025 Mar 10;9(4):781–93. doi: 10.1038/s41562-025-02119-3 (PMC12018265; doi:10.1038/s41562-025-02119-3)
Supplement: Supplementary file 2 — Reporting Summary [file 41562_2025_2119_MOESM2_ESM.pdf]

nature portfolio

Corresponding author(s): Nanthia Suthana

Last updated by author(s): 12/23/2024

Reporting Summary

Nature Portfolio wishes to improve the reproducibility of the work that we publish. This form provides structure for consistency and transparency in reporting. For further information on Nature Portfolio policies, see our Editorial Policies and the Editorial Policy Checklist.

Statistics

For all statistical analyses, confirm that the following items are present in the figure legend, table legend, main text, or Methods section.

n/a	Confirmed	
☐	☒	The exact sample size (*n*) for each experimental group/condition, given as a discrete number and unit of measurement
☐	☒	A statement on whether measurements were taken from distinct samples or whether the same sample was measured repeatedly
☐	☒	The statistical test(s) used AND whether they are one- or two-sided<br>*Only common tests should be described solely by name; describe more complex techniques in the Methods section.*
☐	☒	A description of all covariates tested
☐	☒	A description of any assumptions or corrections, such as tests of normality and adjustment for multiple comparisons
☐	☒	A full description of the statistical parameters including central tendency (e.g. means) or other basic estimates (e.g. regression coefficient) AND variation (e.g. standard deviation) or associated estimates of uncertainty (e.g. confidence intervals)
☐	☒	For null hypothesis testing, the test statistic (e.g. *F*, *t*, *r*) with confidence intervals, effect sizes, degrees of freedom and *P* value noted<br>*Give P values as exact values whenever suitable.*
☒	☐	For Bayesian analysis, information on the choice of priors and Markov chain Monte Carlo settings
☒	☐	For hierarchical and complex designs, identification of the appropriate level for tests and full reporting of outcomes
☐	☒	Estimates of effect sizes (e.g. Cohen's *d*, Pearson's *r*), indicating how they were calculated

Our web collection on statistics for biologists contains articles on many of the points above.

Software and code

Policy information about availability of computer code

Data collection	Neuropace RNS System; Unity game engine (version 2021.2.19f1); OptiTrack MOTIVE (version 3.0)
Data analysis	MATLAB R2021b (The MathWorks, Natick, MA, USA); Signal Processing Toolbox for MATLAB R2021b; Deep Learning Toolbox for MATLAB R2021b; Statistics and Machine Learning Toolbox for MATLAB R2021b; DSP System Toolbox for MATLAB R2021b; Financial Toolbox for MATLAB R2021b; Wavelet Toolbox for MATLAB R2021b; BOSC toolbox for MATLAB (eBOSC)

For manuscripts utilizing custom algorithms or software that are central to the research but not yet described in published literature, software must be made available to editors and reviewers. We strongly encourage code deposition in a community repository (e.g. GitHub). See the Nature Portfolio guidelines for submitting code & software for further information.

Data

Policy information about availability of data

All manuscripts must include a data availability statement. This statement should provide the following information, where applicable:

- Accession codes, unique identifiers, or web links for publicly available datasets
- A description of any restrictions on data availability
- For clinical datasets or third party data, please ensure that the statement adheres to our policy

The data supporting the findings of this study are openly available via Zenodo at https://doi.org/10.5281/zenodo.13743052.

Research involving human participants, their data, or biological material

Policy information about studies with human participants or human data. See also policy information about sex, gender (identity/presentation), and sexual orientation and race, ethnicity and racism.

Reporting on sex and gender	Three male and two female participants took part in this study. Recruitment of participants from both male and female sex was part of the study design. Sex- and gender-based analyses were not performed, given the small cohort size after subdividing by sex and gender. Additionally, sex- and gender-based analyses were not pursued, given that there was no a priori hypothesis of a difference in spatial navigation or episodic memory neurophysiology across sex or gender. As such, results apply to both male and female sex.
Reporting on race, ethnicity, or other socially relevant groupings	Recruitment of participants aimed at representing the demographics in the greater Los Angeles area. In our study and analyses, we do not discriminate participants by race, ethnicity, or other socially relevant groupings.
Population characteristics	Five participants (24-40 years old; three males, two females) who had been chronically implanted with the FDA-approved RNS System (NeuroPace, Inc.) for the treatment of pharmaco-resistant focal epilepsy volunteered for this study. The electrode placements were determined exclusively by clinical treatment criteria. More details are provided in Extended Data Table 1.
Recruitment	Participants were recruited via phone or e-Mail from a database of the University of California Los Angeles, University of California San Francisco, and Staford University.
Ethics oversight	All participants volunteered for the study by providing informed consent according to a protocol approved by the UCLA Medical Institutional Review Board (IRB).

Note that full information on the approval of the study protocol must also be provided in the manuscript.

Field-specific reporting

Please select the one below that is the best fit for your research. If you are not sure, read the appropriate sections before making your selection.

☒ Life sciences ☐ Behavioural & social sciences ☐ Ecological, evolutionary & environmental sciences

For a reference copy of the document with all sections, see nature.com/documents/nr-reporting-summary-flat.pdf

Life sciences study design

All studies must disclose on these points even when the disclosure is negative.

Sample size	Five participants (24-40 years old; three males, two females) with pharmaco-resistant focal epilepsy volunteered for this study. This sample size was chosen, and the experimental procedure was performed individually for each participant to enable data analyses not only on the group level (across all recording channels from all participants) but also independently for each participant in order to investigate the consistency of effects and their reliability across different participants. Further, the sample size selected in this study is comparable to prior studies reporting similar effects in intracranial EEG recordings in freely moving humans.
Data exclusions	The data from all participants was used for data analyses. Each participant had four recording channels. Across all participants, a total of 18 channels were located in MTL regions, including the hippocampus, perirhinal cortex, parahippocampal cortex, and subiculum. Recording channels outside of the MTL were excluded from the main analyses.
Replication	The experimental procedure was repeated five times independently with five different participants. All methods used to perform this study and analyses needed to replicate the presented findings are detailed in the Methods section of the manuscript. In the manuscript, we provide several analyses that show that the effect is strongly present and strikingly consistent across each individual participant that we have tested, suggesting that our results can be generalized beyond the tested sample and reproduced with new and different datasets.
Randomization	All participants were tested with the same experimental protocol with no separate experimental groups. The task comprised real-world and imagined navigation on two distinct spatial routes. Whether participants imagined navigating on their previous or upcoming route in the second or third recording block was counterbalanced.
Blinding	We have tested a rare group of participants with pharmacoresistant epilepsy who have been previously implanted with the NeuroPace RNSSystem for the treatment of their epilepsy. As such, all experiments were aware of this and not blinded with regard to the participants'condition.

Reporting for specific materials, systems and methods

We require information from authors about some types of materials, experimental systems and methods used in many studies. Here, indicate whether each material, system or method listed is relevant to your study. If you are not sure if a list item applies to your research, read the appropriate section before selecting a response.

Materials & experimental systems

n/a	Involved in the study
☒	☐ Antibodies
☒	☐ Eukaryotic cell lines
☒	☐ Palaeontology and archaeology
☒	☐ Animals and other organisms
☒	☐ Clinical data
☒	☐ Dual use research of concern
☒	☐ Plants

Methods

n/a	Involved in the study
☒	☐ ChIP-seq
☒	☐ Flow cytometry
☐	☒ MRI-based neuroimaging

Plants

Seed stocks

Report on the source of all seed stocks or other plant material used. If applicable, state the seed stock centre and catalogue number. If plant specimens were collected from the field, describe the collection location, date and sampling procedures.

Novel plant genotypes

Describe the methods by which all novel plant genotypes were produced. This includes those generated by transgenic approaches, gene editing, chemical/radiation-based mutagenesis and hybridization. For transgenic lines, describe the transformation method, the number of independent lines analyzed and the generation upon which experiments were performed. For gene-edited lines, describe the editor used, the endogenous sequence targeted for editing, the targeting guide RNA sequence (if applicable) and how the editor was applied.

Authentication

Describe any authentication procedures for each seed stock used or novel genotype generated. Describe any experiments used to assess the effect of a mutation and, where applicable, how potential secondary effects (e.g. second site T-DNA insertions, mosiacism, off-target gene editing) were examined.

Magnetic resonance imaging

Experimental design

Design type

MRI was used only to determine the localization of electrode contacts within the brain.

Design specifications

MRI was used only for electrode contact localization; thus, participants did not perform an experimental task during MRI scanning.

Behavioral performance measures

No behavioral performance measures were acquired or derived, since participants did not perform an experimental task during MRI scanning.

Acquisition

Imaging type(s)

structural

Field strength

3 Tesla

Sequence & imaging parameters

standard T1- and T2-weighted sequences

Area of acquisition

whole-brain

Diffusion MRI ☐ Used ☒ Not used

Preprocessing

Preprocessing software

MRI data were preprocessed using FSL (FMRIB Software Library, Oxford University, UK; v5.0.11) for image registration with the FLIRT function (default parameters), and ITK-SNAP (version 3.8.0) for visualization and manual segmentation of electrode contacts.

Normalization

MRI images were not normalized.

Normalization template

MRI images were not normalized.

Noise and artifact removal

No noise or artifact removal procedures were applied.

Volume censoring

Volume censoring was not applied.

Statistical modeling & inference

Model type and settings	No model-based analyses were performed using MRI data.
Effect(s) tested	MRI was used only for electrode contact localization; thus, no task- or stimulus-related analyses were performed using MRI data.

Specify type of analysis: ☒ Whole brain ☐ ROI-based ☐ Both

Statistic type for inference (See Eklund et al. 2016)	No statistical analyses were performed using MRI data.
Correction	No statistical analyses were performed using MRI data; thus, no correction methods were applied.

Models & analysis

n/a	Involved in the study
☒	☐ Functional and/or effective connectivity
☒	☐ Graph analysis
☒	☐ Multivariate modeling or predictive analysis
